# Fighting Bleb Fibrosis After Glaucoma Surgery: Updated Focus on Key Players and Novel Targets for Therapy

**DOI:** 10.3390/ijms26052327

**Published:** 2025-03-05

**Authors:** Matteo Sacchi, Davide Tomaselli, Maria Ludovica Ruggeri, Francesca Bianca Aiello, Pierfilippo Sabella, Stefano Dore, Antonio Pinna, Rodolfo Mastropasqua, Mario Nubile, Luca Agnifili

**Affiliations:** 1 Ophthalmology Unit, Azienda Ospedaliero-Universitaria di Sassari, 07100 Sassari, Italy; matteosacchi.hsg@gmail.com (M.S.);; 2 Ophthalmology Clinic, Alessandro Manzoni Hospital, ASST Lecco, 23900 Lecco, Italy; 3 Department of Neuroscience, Imaging and Clinical Science, “G. d’Annunzio” University of Chieti-Pescara, 66100 Chieti, Italy; 4 Retina Division, Wilmer Eye Institute, Johns Hopkins Hospital, Baltimore, MD 21287, USA; 5 Department of Innovative Technologies in Medicine and Dentistry, “G. d’Annunzio” University of Chieti-Pescara, 66100 Chieti, Italy; francesca.aiello@unich.it; 6 Ophthalmology Clinic, Department of Medicine and Aging Sciences, “G. d’Annunzio” University of Chieti-Pescara, 66100 Chieti, Italyl.agnifili@unich.it (L.A.)

**Keywords:** glaucoma filtration surgery, filtration bleb fibrosis, surgical failure, extra-ocular fibrosis, antifibrotic drugs

## Abstract

Filtration bleb (FB) fibrosis represents the primary risk factor for glaucoma filtration surgery (GFS) failure. We reviewed the most recent literature on post-GFS fibrosis in humans, focusing on novel molecular pathways and antifibrotic treatments. Three main literature searches were conducted. First, we performed a narrative review of two models of extra-ocular fibrosis, idiopathic pulmonary fibrosis and skin fibrosis, to improve the comprehension of ocular fibrosis. Second, we conducted a systematic review of failed FB features in the PubMed, Embase, and Cochrane Library databases. Selected studies were screened based on the functional state and morphological features of FB. Third, we carried out a narrative review of novel potential antifibrotic molecules. In the systematic review, 11 studies met the criteria for analysis. Immunohistochemistry and genomics deemed SPARC and transglutaminases to be important for tissue remodeling and attributed pivotal roles to TGFβ and M2c macrophages in promoting FB fibrosis. Four major mechanisms were identified in the FB failure process: inflammation, fibroblast proliferation and myofibroblast conversion, vascularization, and tissue remodeling. On this basis, an updated model of FB fibrosis was described. Among the pharmacological options, particular attention was given to nintedanib, pirfenidone, and rapamycin, which are used in skin and pulmonary fibrosis, since their promising effects are demonstrated in experimental models of FB fibrosis. Based on the most recent literature, modern patho-physiological models of FB fibrosis should consider TGFβ and M2c macrophages as pivotal players and favorite targets for therapy, while research on antifibrotic strategies should clinically investigate medications utilized in the management of extra-ocular fibrosis.

## 1. Introduction

Glaucoma is the first worldwide cause of irreversible vision loss, and so far, the intra-ocular pressure (IOP) reduction has remained the only proven strategy to contain retinal ganglion cell loss [1]. Although medical and laser therapy effectively control the disease in most cases, approximately 5% of patients require surgery to preserve visual function within 10 years of diagnosis [2]. Glaucoma filtering surgery (GFS) is the most exploited surgical approach to lower IOP, by creating a filtration bleb (FB) from Tenon’s capsule and conjunctiva, which allows aqueous humor (AH) to flow from the anterior chamber to the FB through a scleral fistula [3]. The success rate of GFS varies depending on the technique used and the criteria considered. As for trabeculectomy, the gold standard technique, the success rate ranges from 46% to 62% at five years [4].

A thin and loosely arranged bleb wall is associated with low AH outflow resistance and efficient IOP control, while a thick and highly vascularized conjunctiva hinders fluid movement within the FB [5]. Thus, AH flow through the bleb wall depends on conjunctival permeability, which can be progressively compromised by an increase in connective tissue fiber deposition. The comprehension of the mechanisms involved in FB fibrosis is, therefore, of paramount importance in the attempt to improve the outcomes of GFS.

Generally, the fibrosis process involves the overgrowth, hardening, and scarring of tissues induced by excessive extracellular matrix (EM) deposition, which progressively leads to organ function loss [6,7]. It often results from chronic or acute injuries, including hypoxia, drug toxicity, infections, cancer, trauma, and surgery [6,7]. Key fibrosis mechanisms include recruitment and proliferation of fibroblasts, and their conversion to myofibroblasts, which are major EM producers and express α-smooth muscle actin (αSMA) [6,7,8]. An additional pivotal mechanism is the release of transforming growth factor-β (TGFβ) by activated platelets and immune cells, which promotes fibroblast migration, proliferation, myofibroblast conversion, and vascularization on one side and inhibits lymphocyte and epithelial cell proliferation on the other side [6,9,10].

Smad2 and Smad3 have previously been reported to be transcriptional factors with a key role in controlling fibrosis by promoting gene expressions [11]. Therefore, continuous sustained activation of the TGFβ/Smad2–3 pathway is required in all types of fibrotic processes [12].

To date, the mechanisms responsible for FB fibrosis are not fully understood. Several factors may contribute to post-GFS bleb-wall fibrosis, many of them being related to pre- and post-operative ocular surface alterations and intraoperative tissue manipulations [13,14]. Medical management of post-surgical FB fibrosis involves the use of topical steroids and the administration of intra- and post-operative antifibrotic agents, which, after decades, have not significantly changed [15].

In fact, the most common antifibrotic agents are the antimetabolites 5-fluorouracil (5-FU) and mitomycin C (MMC), which have been in use for over four decades [16]. MMC, due to direct DNA damage, is more effective than 5-FU in inhibiting cell proliferation by intercalating DNA [17,18], providing better long-term IOP control, but it can cause adverse events like FB leakage, blebitis, endophthalmitis, and ocular hypotony [15,19]. Moreover, MMC and 5-FU cannot guarantee adequate control of all mechanisms involved in fibrosis and, thus, long-term preservation of FB function [20,21]. Monoclonal antibodies have been explored as alternatives to MMC or 5-FU. In vivo studies have demonstrated that TGFβ is involved in the processes controlling ocular wound healing [22,23], and therefore a specific anti-TGFβ2 monoclonal antibody (CAT-152) was developed and investigated in a randomized controlled trial (RCT). Unfortunately, it proved ineffective in reducing fibrosis [24]. It is still unclear whether anti-VEGF agents like bevacizumab and ranibizumab may be as effective as MMC in preventing fibrosis after GFS, although they could be useful in neovascular forms [25,26].

This paper reviews the most recent literature on post-GFS fibrosis, aiming to (i) provide a focus on the known key molecular pathways and genomics, (ii) unravel new potential players and pathways, and (iii) suggest new therapeutic targets and molecules.

## 2. Methods

Three main topics related to fibrosis were the object of this search.

**(i) Main features of extra-ocular fibrosis:** Though fibrosis processes differ between organs, we provided a glimpse into the main mechanisms involved in extra-ocular fibrosis to obtain indirect information for the eye. Thus, we briefly analyzed skin fibrosis and idiopathic pulmonary fibrosis (IPF), as they represent two, somehow opposite, of the most important models of fibrosis. The search terms included idiopathic pulmonary fibrosis, IPF, and skin fibrosis. The analysis included reviews, systematic reviews, and meta-analyses.

**(ii) Main features of post-GFS fibrosis:** Literature regarding fibrosis and glaucoma surgery was searched until March 2024 using the PubMed, EMBASE, and Cochrane library databases. Two independent researchers (DT and MLR) performed the search, selection, data extraction, and synthesis and jointly verified the adherence of publications to the topics of this review. Multiple rounds (title, abstract, full text) were used to refine the study selection. Reconciliation procedures, including discussions and a third reviewer, resolved divergent decisions. Studies were not restricted by date or language, and full texts of the articles were screened. We considered the following terms: glaucoma AND fibrosis AND immunohistochemistry; glaucoma AND fibrosis AND genomic; glaucoma AND fibrosis AND biopsy AND filtration bleb. To be included in the analysis, clinical studies had to meet the following criteria: (a) study design: the analysis was not restricted by study design, and case reports, case series, observational studies, and RCTs were included; (b) study participants: patients with glaucoma who had undergone previous GFS; (c) methodology of sample examination: immunohistochemical or genomic analyses; (d) target tissue: conjunctiva, sclera, or Tenon’s capsule; (e) functional state of the FB: functioning vs. non-functioning, or fibrotic vs. non-fibrotic, had to be specified. The decision-making process for determining study eligibility involved tabulating the characteristics of the study intervention. The entities in the research included sample size (e.g., *n* = 10 eyes), demographic data (age, ethnicity), glaucoma phenotype (e.g., primary open-angle glaucoma), type of surgical intervention (e.g., trabeculectomy), use of antimetabolites (e.g., MMC), surgical outcomes (e.g., surgical failure), “surgical failure” definition, FB status (e.g., fibrotic), tissue sampled (e.g., conjunctiva), time from surgery to sampling, inclusion of a control group, molecular targets (e.g., IL-6), type of analysis (e.g., immunohistochemistry), and the resulting outcomes. Metadata, such as authors, journals, and year of publication, were included. Missing information was listed as “not reported”. For each study, key characteristics were compiled and summarized into tables, which were also used to explore possible causes of heterogeneity among the study results. We adhered to the PRISMA 2020 guidelines [27].

**(iii) New investigated treatments to fight post-GFS fibrosis:** We narratively reviewed literature on novel potential antifibrotic molecules proposed to contain post-GFS fibrosis, none of which have currently been tested in humans. Search terms included nintedanib AND glaucoma, pirfenidone AND glaucoma, and rapamycin AND glaucoma. The analysis included in vitro and animal studies.

This study adheres to the ethical principles outlined in the Declaration of Helsinki.

## 3. Results

**Figure 1:**
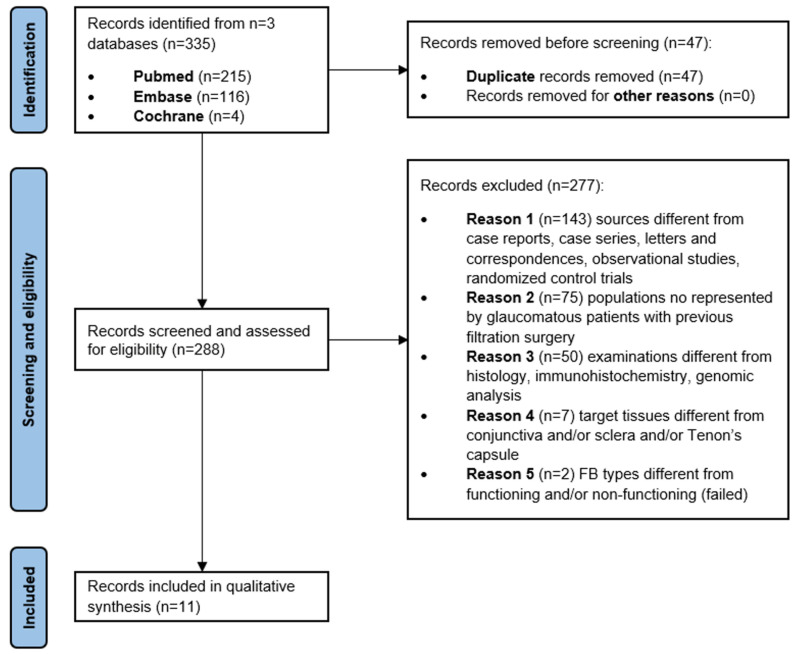
PRISMA diagram of the systematic literature review process. From 3 different databases, 335 records were identified. After removing duplicates, 288 items were selected. Then, 277 records were excluded because of inclusion criteria; thus, 11 records were included in the qualitative synthesis.

A total of 335 publications met the criteria of the systematic review for the second topic. After removing 47 duplicates, 288 publications were screened and assessed for eligibility. Two hundred seventy-seven out of 288 papers were excluded as they did not match the selection criteria; 11 studies were finally included in the analysis (Figure 1).

In more detail, nine studies were conducted by utilizing immunohistochemical analyses [28,29,30,31,32,33,34,35,36], with some of them also applying Northern/Western blot and real-time polymerase chain reaction analyses on human tenon fibroblasts [29,32] or conjunctival fibroblasts [37]. The remaining two studies exclusively concerned genomic aspects [37,38], with one of them performing a genome-wide RNA-sequencing study on human conjunctival fibrosis [37]. All studies considered at least one sample of patients with non-functioning or fibrotic FB, or human tenon fibroblast cell lines; two of them did not have controls [30,36] (Table 1 and Table 2).

A total of 188 and 149 papers were analyzed, respectively, for the “Extra-ocular fibrosis” and the “Proposed therapies” sections of our work.

## 4. Discussion

### 4.1. Main Features of Extra-Ocular Fibrosis

Fibrotic processes occur in different ways, according to the structure and the microenvironment where the organ lies. Nevertheless, we hypothesize that exploring, although not in deep detail, the nature of fibrotic processes in some extra-ocular organs may give some useful insights to better comprehend the mechanisms responsible for post-GFS fibrosis [37].

To test this hypothesis, we considered two opposite models of fibrosis: skin and lung fibrosis. Skin fibrosis is characterized by a self-limiting mechanism triggered by inflammatory stimuli [39,40], whereas IPF is a form of progressive unremitting fibrosis driven by the coexistence of senescence and apoptosis. Notably, IPF is the first pathology for which antifibrotic drugs have been developed and approved by the Food and Drug Administration (FDA) [41,42].

#### 4.1.1. Skin Fibrosis

Skin and the ocular surface show a closely intertwined relationship, presenting common features in immunohistology, steroidogenic properties, and responses to allergic stimuli. Moreover, in terms of embryological development, the conjunctiva is regarded as modified skin [43,44].

Skin wound healing following traumatic injuries comprises three overlapping phases: inflammation (early phase), proliferation, and remodeling (late phases) [39,40]. Pathological wound healing patterns such as hypertrophic scars and keloids are frequent, particularly following burns [40]. While hypertrophic scars consist of αSMA positive cells and parallel rows of collagen I, keloids mostly consist of αSMA negative cells, abundant elastin, and non-organized collagen I and III [39,40].

##### Pathogenesis: Early Phase

The inflammatory phase is thought to begin with the activation of resident mast cells, which are attracted early by circulating complement components (C3a and C5a) [39,40,45,46]. Mast cells release cytokines, which recruit neutrophils and monocytes; chymase and tryptase, which increase fibroblast proliferation and myofibroblast conversion; and histamine, which increases collagen expression and the production of growth factors, including the TGFβ1 (which acts via the Smad2/3 pathway) [40,46,47]. Moreover, mast cells express high levels of the IL33 receptor. IL33 is an interleukin whose main role is to act as an alarmin released during acute events [48]. T lymphocytes in hypertrophic scars show Th2 polarization and release the fibrogenic cytokines IL4 and IL13, which is distinctive of this sub-population [40,49]. M1-like macrophages are recruited within 48–96 h and secrete proinflammatory cytokines (TNFα, IL1, IL6, and IL12). Afterwards, type 2 cytokines (IL4, IL13) polarize macrophages toward the M2-like phenotype, which includes different cell sub-populations producing anti-inflammatory, fibrogenic, and angiogenic mediators [40,50,51]. It has been proposed that in wound tissues, M2 macrophages are primarily type M2c, activated and maintained through a positive IL10-mediated feedback loop: M2c releases IL10 and TGFβ, which in turn stimulate macrophages to polarize into more M2c sub-populations [50].

##### Pathogenesis: Late Phase

Calcium signaling plays a pivotal role in fibroblast migration regulating directional cell movement, redistribution of cytoskeletal proteins, and generation of traction force in migrating fibroblasts [52]. Through this mechanism, PDGF gradients, for instance, promote the turning of migrating fibroblasts [53]. In addition, the increase in cytosolic calcium, mediated by histamine, TGFβ, PDGF, and other mediators, supports the conversion of fibroblasts into myofibroblasts [54].

IL4 and IL13 also promote fibroblast chemotaxis, proliferation, and myofibroblast conversion [55]. In normal wound healing, TGFβ and PDGF depletion induces myofibroblast apoptosis [8,40], whereas other mediators, such as PGE2, have been proposed to induce apoptosis of activated fibroblasts [40]. In this context, the persistence of severe inflammation by prolonging cell survival, proliferation, and EM deposition can lead to excessive fibrosis. EM proteins include type I and III collagens, fibronectin, laminin, and other fibrosis-promoting molecules [6,7,8]. EM deposition is regulated by the interplay between metalloproteases and metalloprotease inhibitors, whose activity is differently regulated depending on the microenvironment [6,7], and by different factors such as the mammalian target of rapamycin (mTOR), a serine/threonine kinase that promotes EM deposition [56].

##### Anti-Skin Fibrosis Treatments

Intralesional injections of 5-FU significantly reduce the recurrence rate of fibrotic scars [57], and the intralesional administration of low concentrations of glucocorticoids further improves the effects of 5-FU [57]. A meta-analysis reported that intralesional injection of botulinum toxin type A is more effective for hypertrophic scars and keloids than intralesional injection of corticosteroid or placebo [58].

Rapamycin (sirolimus), a macrolide antibiotic produced by Streptomyces hygroscopicus, has immunosuppressive effects and acts as an inhibitor of mTOR and as a modulator of the profibrotic TGFβ/Smad signaling cascade [56,59,60]. Many studies have demonstrated that rapamycin has a low toxicity profile and is highly effective [61,62]. When applied to cultured normal and keloidal fibroblasts, rapamycin leads to a dose- and time-dependent reduction in the expression of collagen and alpha-smooth muscle actin, and it has been shown to inhibit the deposition of the EM in both laboratory and live settings [63]. Additionally, rapamycin also reduces the overexpression of collagen types I and III in keloidal fibroblasts [64]. In a small RCT with just four participants, 8% rapamycin ointment did not reduce fibrosis compared to placebo over 6 months of follow-up (Clinical Trial NCT04049552). However, rapamycin has not yet been specifically studied for treating keloids, and further research is needed to fully understand its potential role [65]. The evidence to support any type of laser therapy, alone or in combination with other treatments, compared to other therapies is insufficient [66]. The same consideration is valid for other treatments, such as intralesional injection of autologous fat [67], MMC, imiquimod, interferons, quercetin, verapamil, silicone, and the application of pressure [68].

##### Key Features and Therapy Highlights

The skin fibrosis process is mainly driven by mast cells, growth factors (TGFβ1) and M2-like macrophages, which seem to play a key role in the skin fibrosis process. To date, associative intralesional injections of 5-FU and steroids, or botulinum toxin type A in monotherapy, seem to be the most useful treatments. The effects of rapamycin should be further investigated.

#### 4.1.2. Idiopathic Pulmonary Fibrosis

IPF is a progressive disease involving the alveolar and interstitial spaces of the lung, with the formation of epithelial lined cysts (honeycomb cysts) [41,42]. The causes of IPF are unidentified. Although a familiar form of disease is recognized in 5–20% of cases, causative factors remain undetected [69]. Alveolar type (AT) II cells physiologically replace injured ATI cells, while IPF alveolar epithelial cells and fibroblasts exhibit markers of senescence [41,70,71]. Senescent cells are phenotypically heterogeneous, exhibit an abnormal metabolism, and release pro-inflammatory mediators collectively known as senescent associate secretory phenotype (SASP) [41,72,73].

##### Pathogenesis

SASP molecules produced by epithelial cells (TGFβ, IL-6, TNFα, IL-1α, PDGF, CTGF, and metalloproteases) induce fibroblast proliferation and senescence [41,42,72,74,75].

TGFb promotes fibroblast–myofibroblast conversion and EM deposition signaling through calcium-activated K^+^ channels [76]. Importantly, TGFβ upregulates the mTOR-dependent survival of fibroblasts, and thus, senescent fibroblasts are apoptosis-resistant [41,75,77]. TGFβ upregulates the activity of both mTOR complexes (mTORC1 and mTORC2) [77]. mTORC1 promotes glycolysis, serine and glycine synthesis, and transcription of collagen [77,78,79]. It exerts a positive translational effect on collagen synthesis, mediated by the translational initiation factor 4EBP [41,77]. In IPF, the downregulation of the PI3K inhibitor PTEN further enhances mTOR-dependent pathways due to the physiological role of PI3K in activating mTOR [77]. As for innate and immune cells, their role in the pathogenesis of IPF is unclear. Dysfunctional epithelial-mesenchymal interactions may favor inflammation-enhancing fibrosis. To date, therapeutic protocols based on immunosuppressive drugs have not modified IPF progression [41,42,80].

##### Anti-IPF Treatments

The effect of rapamycin on mTOR activity provides a strong rationale for its use in IPF, though in an RCT, rapamycin has not demonstrated its efficacy in reducing fibrosis [81]. However, mTORC2-mediated pathways and the 4EBP-mediated effect of mTORC1 on collagen translation are rapamycin-insensitive. Thus, dual TORC1/2 inhibitors are currently under investigation [77].

Pirfenidone and nintedanib are two antifibrotic agents that have been demonstrated to slow the decline of respiratory function, but without reverting or arresting the disease progression [41,42,80,82,83]. They were approved for patient care by the FDA in 2014, and their common physio-pathological mechanism entails TGFβ interference [41,82,83], counteracting inflammation and senescence [75,84].

The mechanism of action of pirfenidone in the treatment of idiopathic pulmonary fibrosis (IPF) remains not fully understood. Pirfenidone reduces oxidative stress indicators and inhibits the proliferation of pulmonary fibroblasts and their transformation into myofibroblasts by modulating essential TGFβinduced signaling pathways (such as Smad3, p38, and Akt). It also lowers the expression of TGFβ-induced heat-shock protein 47 (HSP47), which is crucial for procollagen processing and secretion, and decreases the levels of αSMA and type I collagen [41,82,83].

Nintedanib is an intracellular inhibitor of the receptor tyrosine kinases PDGFR, FGFR, and VEGFR, as well as non-receptor tyrosine kinases of the Src family. It suppresses fibroblast proliferation, migration, and contraction; inhibits TGFβ-induced collagen deposition and the transformation of fibroblasts to myofibroblasts; and prevents the polarization of M2 macrophages [41,82,83].

Preclinical and clinical studies to identify other agents able to arrest IPF progression are ongoing. Phase I and phase II RCTs have been performed for the most promising molecules (i.e., pentraxin, monoclonal antibodies recognizing CTGF, src-kinase inhibitors, dasatinib plus quercetin) [75,80,82,83], but phase III RCT results are not yet available.

##### Key Features and Therapy Highlights

The lung fibrosis process is mainly driven by SASP molecules, such as TGFβ, produced by epithelial cells. Dysfunctional epithelial–mesenchymal interactions may enhance fibrosis, favoring the inflammation process. To date, pirfenidone and nintedanib are the only drugs that have demonstrated antifibrotic effects trough TGFβ interference.

### 4.2. Main Features of Post-GFS Fibrosis

#### 4.2.1. Existing Model of Filtration Bleb Fibrosis

A sequence of events leading to fibrosis after GFS has been recently proposed by Peng Tee Khaw et al. [85]. This sequence considers (A) pre-operative risk factors, such as previous ocular surgery, ocular inflammation, glaucoma phenotype (neovascular glaucoma), use of topical medications, patient’s age (childhood), West African ethnicity; (B) surgical manipulation, the new artificially created AH outflow pathway, and the suture material, which promote the release of blood constituents, such as cytokines and growth factors, from the AH, Tenon’s capsule, and sclera; (C) the fibrin clot, which induces an infiltration of leukocytes and platelets at the FB site; (D) fibroblast activation, elongation, and proliferation, with subsequent deposition of EM; (E) tractional forces, growth factors, and the endothelial dysfunction, which promote this process, leading to FB vascularization; and (F) these mechanisms, which negatively affect AH outflow around the surgical flap and within the FB wall, resulting in fibrosis and surgical failure.

#### 4.2.2. An Updated Model of FB Fibrosis

Based on the model proposed by Peng Tee Khaw and coworkers [85], with the aim of implementing and facilitating its interpretation in an updated fashion, we carried out a literature search strictly limited to human studies that included immunohistochemical or genomic analyses of bleb tissue. Based on the articles included in the analysis, we present a patho-physiological dysfunctional model for FB fibrosis, structured into four macro areas: (i) inflammation (stage C and D of the Peng Tee Khaw model), (ii) proliferation of fibroblasts and myofibroblast conversion (stage E), (iii) filtration bleb vascularization (stage E), and (iv) tissue remodeling (stage F) (Table 3).

(i) Inflammation: humoral and cellular response

Plasma proteins promote the migration of neutrophils, macrophages, and lymphocytes to the surgical site, which initiates the inflammatory phase [85]. Immunohistochemical studies have shown that mast cells [31], macrophages [36], and myofibroblasts [28,33], on the cellular side, and TGFβ [33] and interleukin 6 (IL6) [35], on the humoral side, are the main players involved in the inflammatory process following FB formation. Furthermore, genetic studies have shown that many elements are involved in the inflammatory response after GFS: interleukins (IL6 [35,37] and IL13RA2 [38] are upregulated; IL1 [38] and IL33 [37] are downregulated), integrins (ITGA2 [38] and ITGB5 [38] are upregulated), and growth factors (CTGF/CCN2 [38], HGF [38], and AGT [38] are upregulated; IGFBP5 [37] and FGFR3 [37] are downregulated). These alterations suggest the presence of autocrine triggers, which sustain the fibrotic response.

Genomics [37,38] and immunohistochemical studies [29,32,33] documented upregulation of the TGFβ pathway after GFS. TGFβ is upregulated in several eye diseases, such as pterygium, cataract, glaucoma, proliferative vitreoretinopathy, subretinal fibrosis in neovascular age-related macular degeneration, proliferative diabetic retinopathy, and orbital fibrosis in Graves’ ophthalmopathy [86]. Of note, in FB fibrosis, effectors downstream the TGFβ pathway exhibit upregulation, in particular the growth factor CTGF/CCN2 [38], and the antiangiogenic factors THBS1 [38] and THBS2 [38]. Moreover, TGFβ pathway antagonists, such as the growth factor NOV/CCN3 [37], which is a negative regulator of CTGF/CCN2, the transcriptional regulator WISP2/CCN5 [37], and the TGFβ superfamily factor BMP7 [38], are downregulated. The central role of TGFβ is also supported by studies carried out on AH, which reported that TGFβ2 [87,88] and THBS [87,88] are upregulated in patients with failed FB after trabeculectomy.

(ii) Proliferation of fibroblasts and myofibroblast conversion

The process known as fibroblast/myofibroblast conversion has been previously described as “the dark force” in ocular wound healing and fibrosis. TGFβ plays a major role in inducing myofibroblast differentiation through the Smad-dependent and the Smad-independent pathways (e.g., mTOR and the phosphoinositide 3-kinase PI3K/Akt pathways) [86].

The first immune–histological evidence documenting the presence of the contractile protein αSMA in failed FBs dates back to 1996 [28]. The presence of αSMA positive cells has also been confirmed in recent studies, thus strengthening the evidence for a pivotal role of myofibroblasts in FB dysfunction [38].

A genome-wide RNA-sequencing study in human conjunctival fibrosis has shown that different genes promoting myofibroblast conversion and proliferation are upregulated during fibrosis: MYOCD, which encodes myocardin, a smooth muscle-specific transcriptional co-activator of serum response factor (SRF); the transcription factor RELB; PPP1R13L, which encodes a RELA-associated inhibitor that decreases p53/TP53 function; and the oncogenes LMO3, MYB, and BIRC3. Conversely, the DUSP5 gene, which negatively regulates members of the MAPK family (associated with fibroblast and myofibroblast proliferation), and the RASSF2 gene (a tumor suppressor gene), are downregulated. Altogether, these data indicate the promotion of proliferative processes and myofibroblast conversion in dysfunctional or failed FB [37].

(iii) Filtration bleb vascularization

There is evidence that FB vascularization occurs following the initial inflammatory phase and undergoes a progressive reduction with the onset of fibrotic phenomena [85]. In a recent study using immune-histochemical analysis of non-functioning FB capsules, Siggel et al. found positive immunoreactivity for vessel-related biomarkers such as adhesion molecule CD31, lymphatic vessel endothelial receptor 1 (LYVE1), and podoplanin [36]. Given that a colocalization of LYVE1 and podoplanin was not identified, this indicated the presence of blood vessels and the absence of lymphatic vessels. These data were in contrast with a similar study, which described an avascular profile in failed FBs, characterized by negative CD31 [34]. A negative CD34 immunoreactivity has been further identified [37], indicating the absence of arterial-type vessels in fibrotic FBs. These conflicting findings reflect the complexity of vascularization within the bleb wall and suggest the need for kinetic analyses.

(iv) Tissue remodeling

The production of EM is a complex phenomenon mediated by different molecules. When the EM deposition is amplified, tractional forces induce distortion of the tissue architecture within the bleb wall [85]. Transglutaminase enzymes capable of cross-linking EM proteins in proteolysis-resistant complexes could play a role in this phase [89]. The expression of tissue transglutaminase (tTgase) and its reaction products fibronectin (FBN) and ε-(γ-glutamyl)-lysine were found to be increased in non-functioning FB and were upregulated by the incubation of TGFβ2 [29]. The secreted matricellular protein acidic cysteine-rich glycoprotein (SPARC) is known to be involved in scar formation and to promote the signaling of growth factors, such as that of TGFβ, via an increased phosphorylation of Smad2–3 [90]. In fibrotic FBs, the expression of SPARC is increased and is enhanced by TGFβ2 and TGFβ1 incubation. Furthermore, fibroblast proliferation and contractility appear increased to a greater amount when incubated with SPARC and TGFβ1 in combination, compared to the incubation with SPARC or TGFβ1 individually [32].

Mahale and collaborators suggested profibrotic myofibroblast activity in failed FBs characterized by increased expression of αSMA and TGFβ, along with a decrease in the expression of proteoglycans (decorin and lumican) and glycosaminoglycans (chondroitin sulphate). Specifically, collagen I (COL1), collagen III (COL3), decorin (DCN), lumina (LMN), and chondroitin sulphate (CS) were significantly decreased in the inner bleb-wall layers compared to the Tenon’s tissue of the control group [33]. These results suggest that specimens taken from failed FBs are characterized by fibrotic phenomena localized in the innermost FB area.

The EM component proteoglycan 4 (PRG4) is a target gene of the serum response factor (SRF) [91]. It binds to Toll-like receptors (TLRs) [92], playing an anti-inflammatory role in downstream signaling pathways, for instance, in downstream IL-6 signaling [93]. In line with the results of gene expression studies, fibrotic conjunctival tissues demonstrated enhanced IL6 expression and diminished PRG4 protein staining compared to nonfibrotic conjunctival tissues [35].

Genomic studies improved our knowledge regarding fibroblast gene expression, showing that in fibrotic cell lines, the SPARC/ON [28], FBN [29], and COL3A1 [38] are upregulated, while PRG4 [35,37], FBLN1 [37], and COL6A6 [37] are downregulated. These findings agree with immunohistochemistry studies [28,29,35,37], although the observation of increased COL3A1 expression is in contrast with decreased COL3 immunoreactivity [33].

Other studies have reported that both over- and underexpression of genes encoding matrix metalloproteinase 3 (MMP3) [38], MMP8 [38], MMP13 [38], TIMP3 [38], LOX [38], and SERPINE1 [38] were upregulated, whereas MMP10 [37], MMP12 [37], MMP24 [37], and SERPINA1 [38] were downregulated. These findings suggest the crucial activity of EM remodeling molecules characterized by specific enzymes.

SERPINE1, also known as plasminogen activator inhibitor-1 (PAI-1), inhibits urokinase plasminogen activator (uPA), an enzyme that cleaves plasminogen and produces plasmin. Plasmin, independently or in synergism with MMPs, facilitates the degradation of ECM [94]. SERPINA1, or alpha1–proteinase inhibitor (A1PI), is an enzyme known to inhibit various proteases. The upregulation of SERPINE1 and the downregulation of SERPINA1 may therefore indicate a profibrotic state [94].

MMP1, MMP2, and MMP3 and tissue inhibitor of metalloproteinase 2 (TIMP2) immunoreactivity were increased in functioning FBs [30]. In another study, MMP9 was found to be overexpressed in functioning FBs and correlated with CD31-positive vessels, while its inhibitor TIMP3 was found to be overexpressed in failed FBs and correlated with a CD31-negative avascular profile [34]. These data suggest that MMPs and their inhibitors are fundamental in the remodeling process of fibrotic tissues following GFS [95].

### 4.3. Focus Update and Proposed Targets

**Figure 2:**
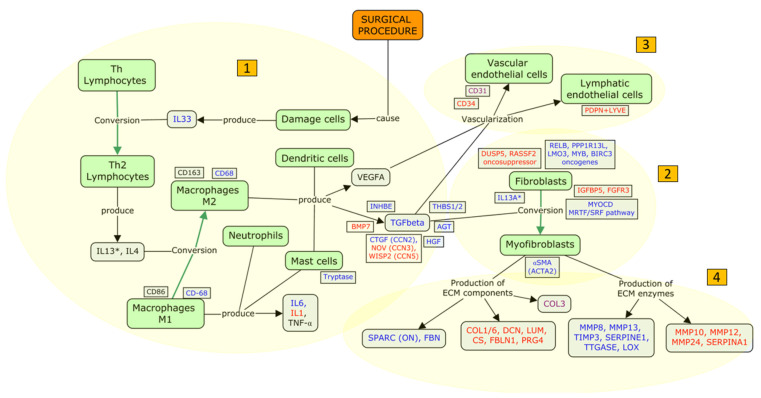
Functional model of ocular fibrosis after glaucoma surgery. The figure aims at connecting the factors and molecules that were found to have a central role in the analyzed studies. Following the surgical procedure (orange), we highlighted central processes (yellow) following surgical procedure potentially involved in surgery failure in hemostasis and fibrin clot formation (1), inflammation (1), proliferation of fibroblasts and myofibroblast conversion (2), filtration bleb vascularization (3), and tissue remodeling (4). In the center of the figure, we represented TGFβ, the key element of the model, with the elements involved in its pathways. We aimed to highlight cells (green), upregulated factors (blue), and downregulated factors (red) that were found to be significantly related to the processes discussed (yellow). Green arrows refer to the main processes of cellular phenotype change. Thus, we tried to connect all the identified elements, despite the presence of discordance evidence (violet). * Refers to IL13 activity on IL13RA expressed by fibroblasts.

Our work aimed to give new insights into the fibrosis model, considering the evidence arising from immunohistochemical or genomic studies, strictly limited to human subjects in post-GFS fibrosis (Figure 2). As previously discussed, the skin and the ocular surface share a closely intertwined relationship, evident in common features such as embryologic development and histological features [43,44]. We have thus considered the skin fibrosis process as a reference for FB fibrosis and for the interaction of cells and molecules in the pathological process. In analogy to the phenomena observed in skin fibrosis, the surgical manipulation during GFS may determine the release of inflammatory mediators, such as IL33, whose main role is to act as an alarmin [48]. Whether, in addition to release, transcriptional regulation affects the production of IL33 is still debated [96].

The activation of type 2 immunity, including Th2 lymphocytes, M2c macrophages, and the molecules IL-13 and IL-4, plays a significant role in the stimulation of profibrotic players (particularly TGFβ) in both the skin [49,55] and the eye [22,29,32,33]. The results of the present review support the fact that TGFβ is a key element involved in post-GFS fibrosis. Increased calcium entry could augment TGFβ-driven fibrosis by enhancing fibroblast activation, ECM production, and NFAT-calcineurin signaling [97].

TGFβ is considered the primary factor that drives fibrosis in many body districts, thus justifying the epithet “the master regulator of fibrosis” [98]. TGFβ is also implicated in ocular diseases, including corneal fibrotic diseases, pterigyum, capsular opacities, proliferative retinal disease [86], and ocular amyloidosis. Amyloidosis, especially familial transthyretin amyloidosis (ATTR), is a significant risk factor for secondary glaucoma, primarily due to aqueous humor outflow obstruction mediated by TGFβ [99]. Patients with amyloidosis-related glaucoma [100] are at a higher risk of surgical failure and may require more advanced interventions [101].

Histological studies have investigated the role of TGFβ1 and TGFβ2 in the GFS-related fibrosis, reporting a greater profibrotic effect of TGFβ1 over TGFβ2, through the stimulation of SPARC production [29,32]. However, the only study that investigated the presence of TGFβ in tissues sampled from failed FB did not distinguish between the two TGFβ isoforms [33]. This evidence, along with the failure of the anti-TGFβ2 monoclonal antibody (CAT-152) [24] in reducing the rate of GFS failure, suggests that other isoforms, such as the β1, should be investigated as potential targets for therapy in humans. TGFβ1-targeted therapies show promise in reducing scar formation after GFS in animal models [102,103].

The TGFβ pathway is primarily dependent on the activation of M2 macrophages, whose differentiation is promoted by fibrogenic cytokines such as IL13 [50,51]. Interestingly, in wound tissues, M2 macrophages are primarily present in the form of the “fibrogenic” type M2c [50]. M2c releases TGFβ, which in turn stimulates macrophages to polarize into more M2c sub-populations [50]. To the best of our knowledge, there are no studies in the literature characterizing the macrophage subtypes (M1, M2a, M2b, and M2c) present in the context of GFS.

### 4.4. New Investigated Treatments to Fight Post-GFS Fibrosis

**Figure 3:**
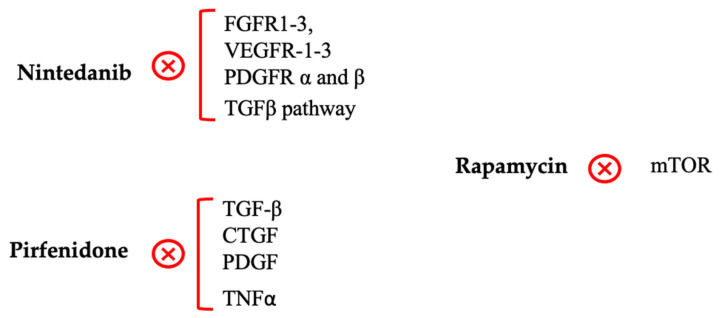
Molecular targets of promising molecules in glaucoma surgery. The figure shows the main molecular pathways involved in the nintedanib, pirfenidone, and rapamycin actions. FGFR1-3: kinase activity of the fibroblast growth factor receptor; VEGFR-1-3: vascular endothelial growth factor receptor; PDGFR: platelet-derived growth factor receptors; TGFβ: transforming growth factor-β; ATP: adenosine triphosphate; CTGF: connective tissue growth factor; PDGF: platelet-derived growth factors; TNFα: tumor necrosis factor-alpha; mTOR: mammalian target of rapamycin; ⊗: Inhibition.

Based on the mechanisms discussed above, we propose novel antifibrotic molecules with the potential to control post-GFS fibrosis, none of which have been tested in humans (Figure 3).

#### 4.4.1. Nintedanib

As previously mentioned, nintedanib and pirfenidone, the first two antifibrotic agents approved by the FDA for IPF, were shown to significantly reduce the rate of respiratory function decline acting on numerous inflammatory targets, including the TGFβ pathway [82,83,104]. These two compounds have been tested in vitro and in animal experimental models of glaucoma.

To date, only one study, conducted in a rabbit model, has evaluated the efficacy of nintedanib in controlling post-GFS fibrosis. In detail, rabbits receiving nintedanib (1 μM) had a better fibrosis score (Indiana Bleb Appearance Grading Scale) compared to the MMC 0.2% group, with no significant side effects [105].

Regarding the safety profile, real-world studies in patients with IPF have highlighted the occurrence of diarrhea, nausea, and vomiting as common adverse events. In clinical trials, serious adverse events, such as bleeding and cardiovascular failure, have been rarely reported, but they frequently led to treatment interruptions and dose adjustments. As a result, in a recent review [106], the authors highlighted the necessity of handling side effects to minimize their impact in clinical practice. The most commonly reported administration route has been the oral capsule. Besides, the ability of this drug to inhibit fibrosis in IPF makes it a central agent in IPF management, with promising evidence that suggests nintedanib as a possible interesting molecule to investigate in patients undergoing GFS.

#### 4.4.2. Pirfenidone

In in vitro studies, pirfenidone reduced the proliferation of fibrotic trabecular meshwork human cells, mitigated the TGFβ2-induced fibrotic markers production (αSMA, F-actin, COL IV, and FBN), and stabilized the cytoskeleton [107]. In a rabbit trabeculectomy model, 0.5% topical pirfenidone significantly reduced scarring and prolonged FB survival.

Moreover, in a different study, the infiltration of inflammatory cells and the proliferation of fibroblasts were significantly lower after treatment with 0.5% and 0.1% pirfenidone compared to controls, with tissue features similar to those found in cases treated with 0.25% MMC. No signs of toxicity were detected at slit lamp, during histological analysis, or in the Draize test in eyes treated with pirfenidone [108]. In a rabbit trabeculectomy model that compared 0.5% pirfenidone, 1% prednisolone acetate, 0.4% MMC, and 10 ng/mL halofuginone, pirfenidone demonstrated a greater efficacy compared to all treatment strategies in suppressing fibroblast proliferation, reducing the number of mononuclear cells and lowering levels of TGFβ, FGF-β, and PDGF immunoreactivity [109]. Moreover, the inhibition of fibroblast and monocyte proliferation was found to be greater in the pirfenidone group than in the MMC-treated group. In an animal model of glaucoma carried out on rabbits, 0.5% pirfenidone showed significant anti-scarring effects and reduced the TGFβ immune-reactivity [110]. Pirfenidone (0.1% and 0.3%) was also compared with MMC (0.05% and 0.1%) and 5-FU (0.2%) on primary cultures of human Tenon’s fibroblasts derived from primary open-angle glaucoma eyes. In this in vitro study, 0.3% pirfenidone inhibited cell migration and reduced αSMA protein expression levels to a greater extent compared to 0.1% MMC, while showing low cytotoxicity [111]. Conversely, 5-FU did not inhibit cell migration and did not reduce αSMA expression.

In the management of pulmonary fibrosis, pirfenidone has been reported to significantly slow respiratory function decline and improve overall survival. Moreover, safety data are promising, though careful management of potential adverse events is still necessary to maximize patient adherence to treatment, which is facilitated by the oral administration route currently used [112].

To summarize, all this evidence suggests that pirfenidone could be an interesting anti-scarring agent to be tested in patients undergoing GFS [113,114,115,116,117].

#### 4.4.3. Rapamycin

Rapamycin, also known as sirolimus, is a macrolide antibiotic that exhibits immunosuppressive properties. It functions as an mTOR inhibitor and modulates the profibrotic TGFβ/Smad signaling pathway [56,59,60].

Interestingly, no serious adverse events related to the use of rapamycin have been reported; however, an increased frequency of infections and cholesterol/triglycerides imbalance have been identified. Different administration routes have been tested (e.g., oral, intraperitoneal, intravenous) [118]. In in vitro studies, rapamycin exhibited a potent inhibition of PDGF-induced human fibroblast proliferation (at 30 ng/mL dose), mostly at the Tenon’s capsule level [119]. Rapamycin was also an effective inhibitor of the fibrotic response induced by TGFβ1 in cultured human conjunctival fibroblast cells [120].

The application of rapamycin in combination with MMC in experimental animal models of GFS was found to efficiently suppress sub-conjunctival scarring [121]. This was further confirmed in a recent study conducted on rabbits undergoing GFS, in which excessive fibroblast proliferation was significantly and safely suppressed by the application of rapamycin at the surgical site [122]. It was also observed that rapamycin is capable of enhancing apoptosis in rabbit fibroblasts by upregulating the expression of caspase 3 and 9 [123]. Similarly, in another study on rabbits, rapamycin demonstrated its effectiveness in preserving FB functionality through the inhibition of fibroblast hyperplasia and the induction of RTFS apoptosis [124]. All these results indicated the effectiveness of rapamycin in suppressing collagen deposits, confirming its potential role in containing fibrosis after GFS (Figure 3).

## 5. Conclusions

GFS is the gold standard treatment for managing medically uncontrolled glaucoma. However, even in well-performed surgeries, there is a risk of GFS failure over time, with FB fibrosis being widely recognized as the main mechanism. Due to the healthcare and socio-economic burden of glaucoma, better control of FB fibrosis is of paramount importance.

To summarize, the overall structure of our work is based on the model of fibrosis described by Peng Tee Khaw and coauthors. Building on this model, we tried to provide some new insights into the underlying mechanisms leading to post-GFS fibrosis and to focus on new possible therapies. To our knowledge, we are the first to propose a framework for cellular and molecular interactions in the context of FB fibrosis.

TGFβ and macrophages certainly play a central role in the complex cascade of fibrosis. M2c macrophages probably act as strong promoters of bleb-wall fibrosis. Based on this hypothesis, we suggest that M2c macrophages could be an interesting target for new anti-scarring approaches. Further studies are needed to better elucidate the role of different isoforms of TGFβ, to characterize macrophage sub-populations, as well as to understand the effects and consequences of inhibiting the TGFβ1-mediated pathways and M2 macrophage activities.

The most widely used antifibrotic agents, 5-FU and MMC, have important limitations. MMC, while providing superior IOP control, is associated with more adverse events, and both agents are insufficient for managing all mechanisms of fibrosis. Among monoclonal antibodies, anti-TGFβ2 (CAT-152) was ineffective in an RCT, while bevacizumab, an anti-VEGF agent, requires further evidence to justify its use as a stand-alone therapy. Among the newer, recently investigated molecules with antifibrotic action, notably nintedanib, pirfenidone, and rapamycin can be promising drugs if the initial observations are confirmed. All three drugs target the TGFβ pathway with specific interactions and have shown efficacy in in vitro studies and animal models, with a high safety profile. RCTs in humans are mandatory to ascertain their tolerability and effectiveness in controlling post-GFS fibrosis.

Among the limitations of our review, the sample baseline characteristics of the included studies showed heterogeneity in age, ethnicity, glaucoma phenotype, surgical technique, use of antimetabolites, definitions of “non-functioning bleb” or “fibrotic bleb”, temporal gaps from first surgery to tissue biopsy, and cell sample culturing. We acknowledge that fibrosis is a complex process involving multiple mechanisms. We established strict criteria for our research and recognize that our results should not be generalized beyond the scope of these criteria. Conversely, a strength of this work is that the systematic review on post-GFS fibrosis is based on selected criteria for the search strategy, and the selection of papers is restricted to clinical studies carried out in human patients who underwent GFS, with available data on FB tissue samples. Moreover, we included a section on “extra-ocular fibrosis”, trying to observe the whole picture of the fibrosis process.

Open Science Framework Registration (currently embargoed until March 31): https://osf.io/4nzu7?view_only=5a541e83f70b42c48505470a775251cb (accessed on 26 February 2025). 
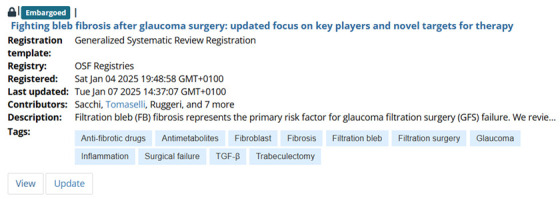


## Figures and Tables

**Table 1:** Characteristics of the included studies by year, author, main and control group features, molecular targets, analysis, and results. Trabe: trabeculectomy; αSMA: alpha smooth muscle actin; tTgase: tissue transglutaminase; FBN: fibrillin1; MMP: matrix metalloproteinase; TIMP 2: tissue inhibitor of metalloproteinases 2; CTGF: connective tissue growth factor, THBS1/2: thrombospondin 1/2, IL13RA2: interleukin 13 receptor subunit alpha 2; HGF: hepatocyte growth factor, AGT: angiotensinogen, CCL11: eotaxin, LOX: lysyl oxidase, ITGA2: alpha-2 integrin, ITGB5: integrin subunit beta 5, COL3A1: pro-alpha1 chains of type III collagen. MYOCD: myocardin, LMO3: LIM domain only 3, IL: interleukin; PRG4: proteoglycan 4; COL6A6 collagen type VI alpha 6 chain; WISP2: WNT1-inducible-signaling pathway protein 2, IGFBP5: insulin-like growth factor-binding protein 5; PPP1R13L: protein phosphatase 1 regulatory subunit 13-like; IHC: immunohistochemistry.

Year	Author	Main Group Features	Control Groups Features	Molecular Targets	Analysis	Results
1996	Mietz et al. [28]	GROUP 1: (n = 7) Bleb tissue (Tenon’s capsule) in eyes with failed trabe + MMC	GROUP 2: (n = 5) Bleb tissue (Tenon’s capsule) in eyes with failed trabe (no MMC/5-FU); GROUP 3: (n = 23) Conjunctiva in non-glaucomatous eyes	αSMA	IHC on slices	+αSMA GROUP 1 vs. GROUP 2, GROUP 1 vs. GROUP 3
2006	Priglinger et al. [29]	GROUP 1: (n = 8) Bleb tissue (Tenon’s capsule) in eyes with failed trabe	GROUP 2: (n = 6) Bleb tissue (Tenon’s capsule) of in vitro functioning trabe models before (GROUP 2a) and after in vitro TGFβ2 treatment (GROUP 2b)	tTgase, FBN, epsilon-gamma-glutamyl-lysine	IHC on slices; RNA/protein study on HTFs with RT-PCR and blot analysis	+tTgase, FBN, epsilon-gamma-glutamyl-lysine GROUP 1 vs. GROUP 2 AND GROUP 2b vs. GROUP 2a
2009	McCluskey et al. [30]	GROUP 1: (n = 10) Bleb tissue (Tenon’s capsule) in eyes with functioning Molteno implant	Not reported	MMP1, MMP2, MMP3, TIMP1, TIMP2, TIMP3	IHC on slices	+MMP1, MMP2, MMP3, TIMP2
2009	Chang et al. [31]	GROUP 1: (n = 8) Peri-bleb tissue (conjunctiva) in eyes with failed trabe	(n = 6) Conjunctiva in medically treated glaucoma (GROUP 2); (n = 7) uveitic glaucoma (GROUP 3); (n = 8) non glaucomatous eyes (GROUP 4)	Mast cells	IHC on slices	+Mast cells GROUP 1 vs. GROUP 4
2011	Fuchshofer et al. [32]	GROUP 1: (n = 3) Bleb tissue (Tenon’s capsule) in eyes with failed trabe	GROUP 2: (n = 3) Tenon’s capsule in non-glaucomatous eyes before (GROUP 2a) and after in vitro TGFβ1 (GROUP 2b), TGFβ2 (GROUP 2c), SPARC (GROUP 2d), SPARC + TGFβ1 (GROUP 2e) treatment	SPARC (secreted acidic cysteine-rich glycoprotein)	IHC on slices; RNA/protein study on HTFs with RT-PCR and blot analysis	+SPARC GROUP 1 vs. GROUP 2a AND GROUP 2b vs. GROUP 2c, +contractility & cells proliferation GROUP 2e vs. GROUP 2d, GROUP 2e vs. GROUP 2b
2015	Mahale et al. [38]	GROUP 1: (n = 7 PCR Array/n = 20 TaqMan) Bleb tissue (Tenon’s capsule) in eyes with failed Ahmed implant	GROUP 2: (n = 2 PCR Array/n = 8 TaqMan) Tenon’s capsule in medically treated glaucomatous eyes	84 selected genes	RNA study on HTFs with RT-PCR; TaqMan assay for 9 selected genes that are >2 fold at PCR	>2 fold: 39 genes. +in ≥5/7: CTGF*, THBS1/2*, INBHE*, IL13RA2*, HGF, AGT, CCL11, MMP1, MMP3*, MMP8, MMP13*, LOX, SERPINE1, ITGA2, ITGB5, αSMA, COL3A1*. -in ≥5/7: IL1A*, BMP7, SERPINA1. Taqman validated: *.
2015	Välimäki et al. [34]	GROUP 1: (n = 3) Bleb tissue (Tenon’s capsule) in eyes with failed implant (Molteno, Baerveldt, Ahmed)	GROUP 2: (n = 3) Tenon’s capsule in eyes with functioning implant (Molteno, Ahmed)	MMP1, MMP2, MMP3, MMP9, TIMP1, TIMP2, TIMP3, CD31	IHC on slices	+TIMP3 GROUP 1, correlate with CD31- avascular profile, +MMP9 GROUP 2, correlate with CD31+ vascular profile
2017	Yu-Wai-Man, Owen, et al. [37]	GROUP 1: (n = 10) Bleb tissue (conjunctiva) in eyes with failed glaucoma surgery	GROUP 2: (n = 7) Conjunctiva in medically treated glaucoma eyes	Genome wide	RNA study on HTFs with RNA-Seq; RT-PCR for 11 selected genes that are >2 fold at NGS	>2 fold up/downregulation: 246 genes. PCR validated: +MYOCD, LMO3, IL6, RELB; -PRG4, CD34, IL33, COL6A6, MMP10, WISP2, IGFBP5. Significant others: +PPP1R13L, MYB, BIRC3; -DUSP5, FGFR3, NOV, FBLN1, MMP12, MMP24, RASSF2
2017	Yu-Wai-Man, Tagalakis, et al. [35]	GROUP 1: (n = 24) Bleb tissue (conjunctiva) in eyes with failed glaucoma surgery (trabe, implants)	GROUP 2: (n = 14) Conjunctiva in medically treated glaucomatous eyes	IL6, PRG4	IHC on slices; RNA study on HCFs with RT-PCR and TaqMan assay	+IL6, -PRG4 GROUP 1 vs. GROUP 2
2017	Mahale et al. [33]	GROUP 1: (n = 14) Bleb tissue (Tenon’s capsule) in eyes with failed Ahmed implant	GROUP 2: (n = 8) Tenon’s capsule in eyes in non-treated glaucomatous eyes	COL1, COL3, DCN, LUM, CS, ACAN, KS, αSMA and TGFβ	IHC on slices	+αSMA, TGFβ, -COL3, DCN, LUM, CS in the inner layers GROUP 1 vs. GROUP 2
2020	Siggel et al. [36]	GROUP 1: (n = 15) Bleb tissue (Tenon’s capsule) in eyes with failed Baerveldt implant	Not reported	CD31, CD68, PDPN (D2-40, Podoplanin), LYVE-1	IHC on slices	+++CD31 and +CD68 in the outer layers, +PDPN in the inner/outer layers, -PDPN AND LYVE1

**Table 2:** Patient demographics, disease, and surgery-related features in the selected studies. POAG: primary open angle glaucoma; PG: phacolytic glaucoma; PXG: pseudoexfoliation glaucoma; ICE: iridocorneal endothelial syndrome; trabe: trabeculectomy; CG: chronic glaucoma; NVG: neovascular glaucoma; UG: uveitis glaucoma; SOAG: secondary open angle glaucoma; MMC: mytomicin C.

Year	Author	Age	Ethnicity	Glaucoma Type	Surgical Technique	Perioperative Antimetabolites	“Failure” Definition (GROUP 1)	Time from Surgery to Biopsy
1996	Mietz et al. [28]	Adults, children	Not reported	POAG, PG, PXG, ICE, aniridia, Peter’s anomaly, trauma	GROUP 1: trabe; GROUP 2: trabe	GROUP 1: MMC; GROUP 2: no	Clinically failed secondary to fibrosis at the level of the episclera or related to the formation of Tenon’s capsule cysts	GROUP 1: 1 weeks–10 months; GROUP 2: 1–11 months
2006	Priglinger et al. [29]	Adults	Not reported	POAG, PXG, CG	GROUP 1: trabe; GROUP 2: trabe	GROUP 1: 3/8 yes; GROUP 2: not reported	Formation of Tenon cysts	7 weeks–2 years
2009	McCluskey et al. [30]	Adults	Not reported	POAG, PXG, NVG, trauma, ghost cells	Molteno implant	Not reported	Not reported	2 months–22.9 years
2009	Chang et al. [31]	Adults	Asian, Afro-Caribbean, Caucasian, Turkish	Specified only for GROUP 3: UG	Trabe	Not reported	Not reported	Not reported
2011	Fuchshofer et al. [32]	Not reported	Not reported	Not reported	Trabe	Not reported	Not reported	Not reported
2015	Mahale et al. [38]	Adults, children	Not reported	Not reported	Ahmed implant	Not reported	Uncontrolled IOP with maximal tolerated medically therapy	GROUP 1 for PCR Array: 12–144 months; GROUP 1 for TaqMan: 6–156 months
2015	Välimäki et al. [34]	Adults	Not reported	POAG, PXG, NVG, UG	Molteno, Baerveldt, Ahmed implants	Not reported	IOP > 21 mmHg or <20% reduction in IOP from baseline with maximal tolerated medication	GROUP 1: 6–108 months; GROUP 2: 31–67 months
2017	Yu-Wai-Man, Owen, et al. [37]	Adults	Asian, Afro-Caribbean, Caucasian	POAG, SOAG, CG	Not reported	Not reported	Not reported	Not reported
2017	Yu-Wai-Man, Tagalakis, et al. [35]	Adults	Asian, Afro-Caribbean, White	Not reported	Trabe, implants	Not reported	Not reported	Not reported
2017	Mahale et al. [33]	Adults, children	Not reported	POAG, SOAG (no NVG), CG	Ahmed implant	Not reported	Intraocular pressure above target levels on maximum medical therapy determined by the treating physician	3–156 months
2020	Siggel et al. [36]	Adults, children	Not reported	POAG, CG, silicon oil, steroid induced, aphakic, ICE	Baerveldt implant	Not reported	Poor function	1.1 months–6.3 years

**Table 3:** Key elements involved in different stages of the bleb fibrosis process. The table shows key elements involved in the bleb fibrosis process evidenced by immunohistochemistry and genetic/genomic studies. Blue indicates upregulated factors, whereas red indicates downregulation. (*) refers to the factors involved in functioning blebs. EM: extracellular matrix.

Processes	Evidences from IHC	Evidences from Genetic/Genomic
Inflammation: humoral and cellular response	Mast cells (tryptase), macrophages (CD68), humoral response (IL6, TGFβ)	Interleukines (IL1, IL6, IL13RA2, IL33), chemokines (CCL11), integrins (ITGA2, ITGB5), growth factors (CTGF/CCN2, HGF, AGT, IGFBP5, FGFR3), TGFβ pathways (CTGF/CCN2, NOV/CCN3, WISP2/CCN5, THBS1, THBS2, INBHE, BMP7)
Proliferation of fibroblasts and myofibroblast conversion	Activity (αSMA/ACTA2)	Activity (αSMA/ACTA2), differentiation (MYOCD), proliferation/apoptosis (DUSP5, RELB, PPP1R13L, LMO3, MYB, BIRC3, RASSF2)
Filtration bleb vascularization	Arterial vessels (CD31, CD31), lymphatic vessels (PDPN + LYVE)	Arterial vessels (CD34)
Tissue remodelling	ECM composition (PRG4, SPARC/ON, FBN, COL1, COL3, DCN, LUM, CS), ECM turnover (MMP1*, MMP2*, MMP3*, MMP9*, TIMP2*, TIMP3)	ECM composition (PRG4, SPARC/ON, FBN, FBLN1, COL3A1, COL6A6), ECM turnover (MMP1, MMP3, MMP8, MMP10, MMP12, MMP13, MMP24, TIMP3, SERPINE1, SERPINA1, LOX)
